# Genetic Diversity of *Salmonella enteric *serovar Typhi and Paratyphi in Shenzhen, China from 2002 through 2007

**DOI:** 10.1186/1471-2180-10-32

**Published:** 2010-01-30

**Authors:** Weiyuan Wu, Hui Wang, Jian Lu, Jinsong Wu, Minjun Chen, Yingchun Xu, Yuemei Lu

**Affiliations:** 1 Department of Laboratory Medicine, Shenzhen People's Hospital, Jinan University Second Clinical Medical College, Shenzhen (Guangdong), 518020, PR China; 2 Department of Clinical Laboratory, Peking Union Medical College Hospital, Chinese Academy of Medical Sciences, Beijing 100730, PR China; 3 Department of Infectious Disease, Shenzhen East Lake Hospital, Shenzhen (Guangdong), 518020, PR China

## Abstract

**Background:**

Typhoid and paratyphoid fever are endemic in China. The objective of this investigation was to determine the molecular features of nalidixic acid-resistant *Salmonella enteric *serovar Typhi (*S. typhi*) and Paratyphi (*S. paratyphi*) from blood isolates in Shenzhen, China.

**Results:**

Twenty-five *S. typhi *and 66 *S. paratyphi *were isolated from 91 bacteriemic patients between 2002 and 2007 at a hospital in Shenzhen, Southern China. Fifty-two percent (13/25) of *S. typhi *and 95.3% (61/64) of *S. paratyphi *A were resistant to nalidixic acid. Sixty-seven isolates of nalidixic acid-resistant *Salmonella *(NARS) showed decreased susceptibility to ciprofloxacin (MICs of 0.125-1 μg/mL). All 75 NARS isolates had a single substitution in the quinolone resistance-determining region (QRDR) of GyrA (Ser83→Phe/Pro/Tyr, or Asp87→Gly/Asn), and 90.7% of these isolates carried the substitution Ser83Phe in GyrA. No mutation was found in the QRDR of *gyrB*, *parC*, or *parE*. Plasmid mediated quinolone resistance genes including *qnr *and *aac(6')-Ib-cr *were not detected in any isolate. Twenty-two distinct pulsed field gel electrophoresis (PFGE) patterns were observed among *S. typhi*. Sixty-four isolates of *S. paratyphi *A belonged to one clone. Eighty-seven investigated inpatients were infected in the community. Six patients infected by *S. paratyphi *A had a travel history before infection.

**Conclusions:**

Nalidixic acid-resistant *S. typhi *and *S. paratyphi *A blood isolates were highly prevalent in Shenzhen, China. PFGE showed the variable genetic diversity of nalidixic acid-resistant *S. typhi *and limited genetic diversity of nalidixic acid -resistant *S. paratyphi *A.

## Background

Typhoid and paratyphoid fever, due to infection with *Salmonella enteric *serovar Typhi (*S. typhi*) and Paratyphi (*S. paratyphi*), are major global problems. Nalidixic acid-resistant (NAR) *S. typhi *and *S. paratyphi *are endemic to many Asian countries [1]. NAR isolates have reduced susceptibility to fluoroquinolones, which is associated with higher rates of morbidity and mortality, particularly prolonged fever clearance time and increased need for retreatment [2]. Quinolone resistance in *Salmonella *is usually associated with mutations of the target site, DNA gyrase, most commonly in the quinolone resistance-determining region (QRDR) of the A subunit. Plasmid mediated quinolone resistance genes of *qnr *(*qnr*A, *qnr*B, *qnr*S, and *qnr*D) and *aac(6')-Ib-cr *has also been described in quinolone-resistant non-Typhi Salmonella[3,4]. In China, the molecular characterization and antimicrobial susceptibility of non-Typhi serotypes of *Salmonella enterica *have been recently reported [4], however the molecular mechanism of resistance and epidemiology of NAR *S. typhi *and *S. paratyphi *is not available. In this study we investigated the molecular basis of resistance and the epidemiology of 25 *S. typhi *and 66 *S. paratyphi *blood isolates that were recovered from hospitalized patients in Shenzhen City, Southern China over 6-year period. The cases were retrospectively examined for epidemiologic analysis.

## Methods

The study site was the Shenzhen People's Hospital, a 1090-bed medical center for patients who reside in Shenzhen, Guangdong Province of Southern China, with an estimated population of 12 million people. This study has been performed with the approval of Ethics committee of Shenzhen People's Hospital (Shenzhen, China).

### Bacterial isolates and susceptibility testing

Ninety-one non-duplicate isolates of Salmonella (25 *S. typhi*, 64 *S. paratyphi *A, 1 *S. paratyphi *B, and 1 *S. paratyphi *C) were consecutively obtained from blood cultures of 91 patients with typhoid or paratyphoid from 2002 through 2007 (2002, n = 13; 2003, n = 27; 2004, n = 21; 2005, n = 6; 2006, n = 15; 2007, n = 9). All isolates were identified with standard biochemical tests and specific antisera (Institute of Biological Products, Lanzhou, China). The MICs of nalidixic acid and the other antimicrobial agents were determined by agar dilution method according to Clinical and Laboratory Standard Institute (CLSI) M7-A7 [5] and were interpreted according to CLSI performance standard M100-S17 [6]. The antimicrobials were supplied and stored according to the manufacturer's instructions. *Escherichia coli *ATCC 25922 and *Pseudomonas aeruginosa *ATCC 27853 were used as quality control strains for susceptibility testing. Multidrug-resistant strains were defined as those resistant to ampicillin, chloramphenicol, and trimethoprim/sulfamethoxazole (TMP-SMZ) [7].

### Polymerase chain reaction (PCR) and DNA sequencing

All 91 isolates were screened for the *qnr *(*qnrA*, *qnrB*, and *qnrS*) genes by multiplex PCR [8] and for *aac(6')-Ib *by PCR [9]. PCR amplification of the quinolone resistance-determining regions (QRDRs) of *gyrA*, *gyrB*, *parC*, and *parE *was performed in all isolates as described previously [10]. Mutations in the *gyrA*, *gyrB*, *parC*, and *parE *genes were identified by DNA sequencing. The PCR products were purified by using a QIAquick PCR purification kit (Qiagen, Hilden, Germany). DNA sequencing of both strands was performed by the direct sequencing method with an ABI Prism 3100 generic analyzer (Applied Biosystems, Foster City, CA), and the DNA sequences of the QRDRs of *gyrA*, *gyrB*, *parC*, and *parE *were compared with the DNA sequences of the QRDRs of *S. typhi, S. paratyphi *A, and *S. paratyphi *B (GenBank: NC_004631, NC_006511, NC_010102). β-lactamase genes were detected by PCR with primers specific for *bla*_CTX-M_, *bla*_TEM_, *bla*_SHV_, *bla*_OXA _among isolates resistant to ampicillin as described previously [11-13], and PCR products were sequenced as described above. Class 1 intergron as was investigated by PCR. PCR products were sequenced using a pair of specific primers of 5'CS and 3'CS for multidrug-resistant isolates [14].

### Pulsed field gel electrophoresis

PFGE of *Xba*I (New England)-digested genomic DNA of all isolates was carried out using the CHEF MAPPER system (Bio-Rad), as described by the standard PulseNet protocol for *Salmonella *species by the Centers for Disease Control and Prevention [15]. Similarities among macrorestriction patterns were determined both by visual comparison and computer matching with BioNumerics 4.0 software. Dendrograms for similarity were built using the unweighted-pair group method using arithmetic averages. Patterns differing by zero to three fragments are considered to belong to the same PFGE type according to the method of Tenover et al [16].

### Case investigation

A case was defined as illness compatible with acute typhoid or paratyphoid fever and isolation of *S. typhi *or *S. paratyphi *from a sterile site. A total of 87 cases of acute *S. typhi *and *S. paratyphi *A infections were retrospectively examined over a 6-year period; the medical records from 2 outpatients infected by *S. paratyphi *A were unavailable. Demographic, epidemiologic, and clinical information were recorded on case report forms that included age, sex, habitation, history of travel in the 30 days preceding illness onset, clinical symptoms and signs, laboratory data, and antimicrobial therapy. We did not include data about previous immunization against typhoid fever because it was unavailable for most of patients. Statistical analysis was performed using SPSS for Windows (release 13.0).

## Results

### Antimicrobials susceptibility

Fifty-two percent (13/25) of *S. typhi *and 95.3% (61/64) of *S. paratyphi *A were resistant to nalidixic acid, respectively (table 1). More than half of nalidixic acid-resistant *S. paratyphi *A isolates were detected between 2003 and 2004 (table 2). Sixty-seven isolates of nalidixic acid-resistant *Salmonella *(including 6 *S. typhi*, 60 *S. paratyphi *A and 1 *S. paratyphi *C) showed decreased susceptibility to ciprofloxacin (MIC = 0.125-1 μg/mL), although all were susceptible to the fluoroquinolones according to current CLSI breakpoints.

**Table 1:** Susceptibilities of *S. typhi *and *S. paratyphi *A to 12 antimicrobial agents

Antimicrobial agents	*S. typhi *(N = 25)				*S. paratyphi *A (N = 64)			
		
	R%	S%	MIC_50_ (μg/mL)	MIC_90_ (μg/mL)	R%	S%	MIC_50_ (μg/mL)	MIC_90_ (μg/mL)
Nalidixic acid	52	48	64	≥256	95.3	4.7	≥256	≥256
Norfloxacin	0	100	0.25	1	0	100	2	2
Ciprofloxacin	0	100	0.064	0.25	0	100	0.5	0.5
Levofloxacin	0	100	0.125	0.5	0	100	1	1
Gatifloxacin	0	100	0.064	0.25	0	100	0.5	1
Sparfloxacin*	-	-	0.125	1	-	-	1	2
Moxifloxacin*	-	-	0.125	0.5	-	-	1	1
Cefotaxime	0	100	0.064	0.064	1.6	98.4	0.125	0.5
Ceftriaxone	0	100	0.064	0.125	1.6	98.4	0.125	0.25
Ampicillin	4	96	1	4	1.6	98.4	2	4
Chloramphenicol	0	100	2	4	0	98.4	4	8
Trimethoprim/sulfamethoxazole	0	100	0.25	0.25	0	100	0.25	0.25

* CLSI had no breakpoints for these agents

R - Resistant

S - Susceptible

**Table 2:** Isolates of NARS collected during 2002 to 2007

Microorganism	2002	2003	2004	2005	2006	2007
*S. typhi*	5 (7)*	2 (7)	1 (3)	1 (1)	1 (3)	3 (4)
*S. paratyphi *A	5 (6)	19 (19)	16 (18)	4 (4)	12 (12)	5 (5)
*S. paratyphi *B	0 (0)	0 (1)	0 (0)	0 (0)	0 (0)	0 (0)
*S. paratyphi *C	0 (0)	0 (0)	0 (0)	1 (1)	0 (0)	0 (0)

* parentheses referring to the total number of isolates collected annually for each species

Twenty-five *S. typhi *and 64 *S. paratyphi *A were highly susceptible to ampicillin, chloramphenicol and TMP-SMZ, with the overall susceptibility being 96%~100% (table 1). Resistance to ceftriaxone and cefotaxime was detected only in 1 isolate of *S. paratyphi *A (MIC = 64 μg/mL). Interestingly, only one *S. typhi *showed resistance to ampicillin (MIC ≥ 256 μg/mL). One isolate of *S. paratyphi *B was susceptible to all drugs tested and one isolate of *S. paratyphi *C showed multiple resistance to nalidixic acid (MIC ≥ 256 μg/mL), ampicillin (MIC ≥ 256 μg/mL), chloramphenicol (MIC ≥ 256 μg/mL), and TMP-SMZ (MIC ≥ 32 μg/mL).

### PCR and DNA sequencing

All 75 NARS had a single point mutation in the QRDR of *gyrA *that led to a single-amino-acid substitution at codons 83 or 87 of GyrA (Ser83→Phe, Ser83→Pro, Ser83→Tyr, Asp87→Gly, or Asp87→Asn) (table 3), and 90.7% (68/75) of these isolates carried the substitution Ser83Phe in GyrA. No mutation was found in the QRDR of *gyrB*, *parC*, or *parE*. For all 16 NASS isolates, no point mutation was detected in the QRDR of *gyrA/B *or *parC/E *gene. Plasmid-mediated quinolone resistance genes including *qnr *and *aac(6')-Ib-cr *were not detected in any isolate. The *bla*_CTX-M-14 _gene was detected in the ceftriaxone-resistant isolate of *S. paratyphi *A, with IS*Ecp1 *located on the upstream of *bla*_CTX-M-14 _gene. A 1.9-kb class 1 integron gene cassette *dhfrXII-orfF-aadA2 *was identified in the multidrug-resistant isolate of *S. paratyphi *C, in which *bla*_TEM-1 _gene was also detected. None of *bla*_CTX-M_, *bla*_TEM_, *bla*_SHV _and *bla*_OXA _genes were identified in the ampicillin-resistant isolate of *S. typhi*.

**Table 3:** The point mutation in the QRDR of *gyrA *of nalidixic acid-resistant *Salmonella*.

Point mutation in the QRDR of *gyrA*	MIC (μg/mL)*	
	
	nalidixic acid	ciprofloxacin
nalidixic acid-resistant *S. typhi*		
Ser83→Phe (TCC→TTC)	≥ 256 (9)	0.06 (4), 0.125 (1), 0.25 (2), 0.5 (2)
Asp87→Gly (GAC→GGC)	128 (1)	0.06 (1)
Asp87→Asn (GAC→AAC)	64 (2), ≥ 256 (1)	0.06 (2), 0.25 (1)
nalidixic acid-resistant *S. paratyphi *A		
Ser83→Phe (TCC→TTC)	≥ 256 (59)	0.25 (8), 0.5 (50), 1 (1)
Ser83→Pro (TCC→CCC)	32 (2)	0.125 (1), 0.03 (1)
nalidixic acid-resistant *S. paratyphi *C		
Ser83→Tyr (TCC→TAC)	≥ 256 (1)	0.125 (1)

* parentheses referring to the number of isolates with the point mutation in the QRDR of *gyrA*

### PFGE

**Figure 1:**
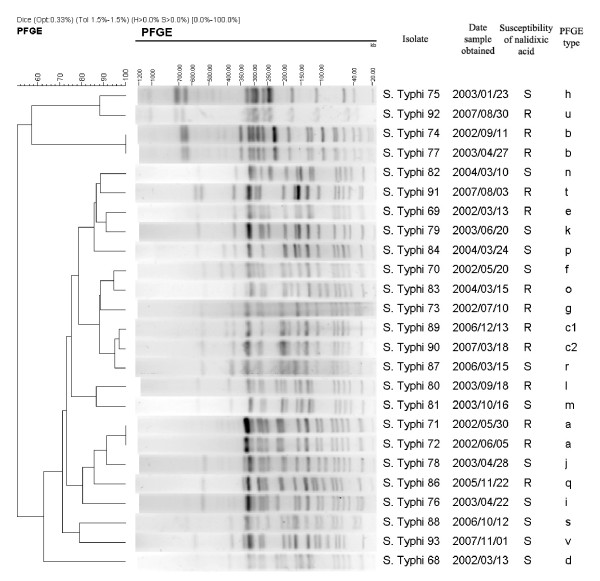
**Dendrogram for the *S. typhi *isolates with distinct PFGE types**. Genetic similarity was calculated by the Dice coefficients. R, Resistant; S, Susceptible.

**Figure 2:**
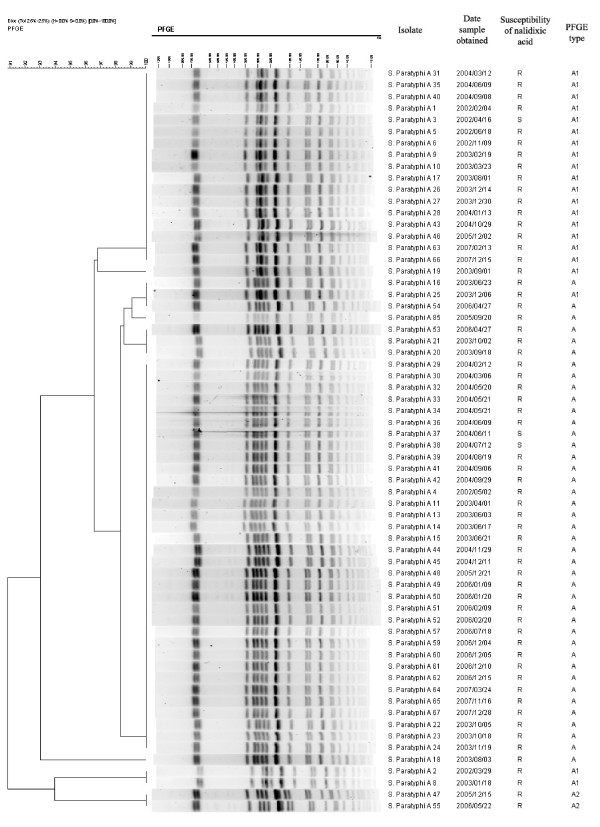
**Dendrogram for the *S. paratyphi *A isolates with the same PFGE types**. Genetic similarity was calculated by the Dice coefficients. R, Resistant; S, Susceptible.

**Figure 3:**
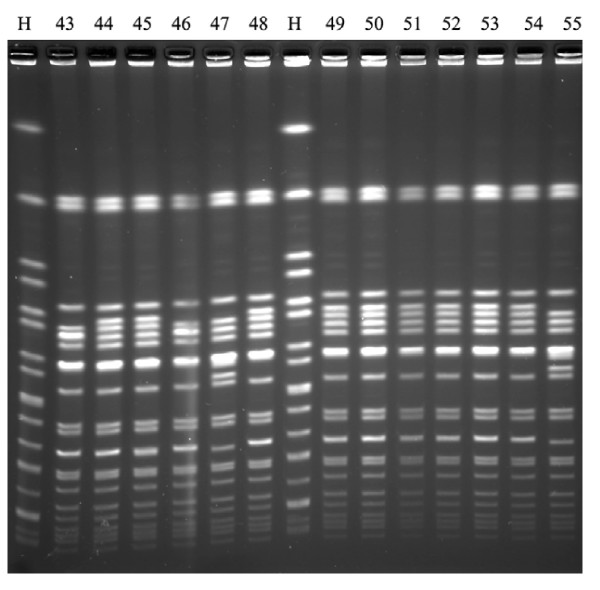
**Analysis of *S. paratyphi *A isolates by PFGE of *Xba*I restriction digests**. H standard strain H9812; isolates 44, 45, 48-54 (PFGE type A); isolates 43, 46 (PFGE type A1); isolates 47 and 55 (PFGE type A2).

Overall, 22 different PFGE patterns were observed among 25 isolates of *S. typhi *from 2002 through 2007 (figure 1); 10 of 22 PFGE patterns were identified among 13 nalidixic acid-resistant isolates. The variable genetic diversity among *S. typhi *isolates suggested endemic disease from multiple sources. In contrast, among 64 isolates of *S. paratyphi *A, 41 isolates (including 39 NARS) were assigned to PFGE type A (figure 2 and 3), 21 isolates (including 20 nalidixic acid-resistant isolates) belonging to subtype A1 (difference by one band of ~310 kb compared to type A), and 2 nalidixic acid-resistant isolates to subtype A2 (difference by one band of ~310 kb and one band of ~190 kb compared to type A). The limited genetic diversity (similarity coefficient of 91%) among *S. paratyphi *A isolates indicated endemic disease from the presence of a single clone over 6-year period.

### Case investigation

Infection was acquired in community in 87 patients. All patients were residents of Shenzhen City, and were mostly young or middle age and lived in sanitary environments. Six patients infected by *S. paratyphi *A had traveled to other cities or regions in the 30 days preceding illness onset, including Shaoguan City in Southern China (n = 1), Chongqing City and Guizhou province in Southwestern China (n = 3), Taiwan (n = 1), and Bangladesh (n = 1). More than 80% of patients (20 *S. typhi*-infected patients and 52 *S. paratyphi *A-infected patients, respectively) had received antimicrobials prior to hospital admission. They were primarily hospitalized due to fever for at least 3 days.

Epidemiological, clinical and laboratory features are presented in table 4. Clinical treatment and outcome in 23 nalidixic acid-susceptible *Salmonella *(NASS) and nalidixic acid-resistant *Salmonella *(NARS)-infected patients treated with fluoroquinolones alone are shown in table 5. The mean fever clearance time for 6 patients infected by NASS and 17 patients infected by NARS were 75.5 hours and 119.2 hours, respectively, *p *= 0.178. The illness of the patients infected by ceftriaxone-resistant *S. paratyphi *A improved after being treated with ciprofloxacin (0.4 g IV q12h) for 11 days. When ceftriaxone was combined with TMP-SMZ (0.96 g PO q12h) this was shortened to 6 days during hospitalization; home therapy continued with oral antimicrobials.

**Table 4:** Epidemiological, clinical and laboratory features in the 87 inpatients with culture-confirmed enteric fever

Parameter^*a*^	*S. typhi*-infected patients (n = 25)	*S. paratyphi *A-infected patients (n = 62)
Mean age (yr) (range)	26.7 (0-67)	32.7 (16-62)
Male	14 (56)	36 (58)
Previous enteric fever	0 (0)	1 (2)
Contact with patients with enteric fever	0 (0)	1 (2)
Insalubrity intake	3 (12)	1 (2)
Sanitary latrine	25 (100)	61 (98)
History of travel in the 30 days preceding illness onset	0 (0)	6 (10)
Symptoms		
Fever	25 (100)	61 (98)
Headache	9 (36)	31 (50)
Chills	18 (72)	45 (73)
Rigor	5 (20)	15 (24)
Sweating	4 (16)	9 (15)
Cough	3 (12)	13 (21)
Abdominal pain	3 (12)	7 (11)
Nausea	3 (12)	10 (16)
Vomiting	2 (8)	8 (13)
Diarrhea	2 (8)	12 (19)
Myalgia	6 (24)	11 (18)
Weight loss	8 (32)	9 (15)
Erythra	3 (12)	3 (5)
Physical finding		
Abdominal tenderness	2 (8)	7 (11)
Hepatomegaly	3 (12)	11 (18)
Splenomegaly	7 (28)	30 (48)
Laboratory finding		
Mean WBC count (× 10^9^/L) (range)	5.6 (2.1-8.5)	5.4 (1.0-16.8)
positive Widal test	16 (84)^*b*^	9 (16)^*c*^
ALT (> 40 IU/L)^*d*^	18 (72)	46 (74)
AST (> 45 IU/L)^*e*^	17 (68)	45 (73)
Complications^*f*^	6 (24)	13 (21)

*a *Data are presented as no. (%).

*b *Only 19 patients were detected.

*c *Only 55 patients were detected.

*d *AST, aspartate transaminase (normal range, 0-40 IU/L).

*e *ALT, alanine transaminase (normal range, 0-45 IU/L).

*f *including toxic hepatitis, toxic myocarditis, intestinal hemorrhage, bronchitis, pneumonia, and bacterial meningitis.

**Table 5:** Clinical treatments and outcomes in nalidixic acid-susceptible *Salmonella *(NASS) and nalidixic acid-resistant *Salmonella *(NARS)-infected patients treated with fluoroquinolones only^*a*^

Antimicrobial agents		NASS-infected patients (n = 6)		NARS-infected patients (n = 17)	
			
	Dosage	Number	Duration (d)	Number	Duration (d)
Ciprofloxacin	0.4 g IV q12h	5	7~13	8	7~21
	0.2 g IV q12h	1	5	2	10~15
Levofloxacin	0.3 g IV q12h	-	-	1	7
	0.2 g IV q12h	-	-	2	7~8
Gatifloxacin	0.2 g IV q12h	-	-	3	10~14
	0.4 g IV q24h	-	-	1	13

^*a*^All of these 23 patients treated with fluoroquinolones only were cured.

## Discussion

Nalidixic acid-resistant *S. typhi *and *S. paratyphi *are endemic in Vietnam and some other South Asia countries such as India, Pakistan, Bangladesh, and Nepal [17], with a resistance rate range of 38-97%. It has been reported that more than 70% of *Salmonella *enteric serovar Typhimurium isolates are resistant to ciprofloxacin and some have become multidrug-resistant in regions of China [4]. In this study, 52% of *S. typhi *and 95% of *S. paratyphi *A showed resistance to nalidixic acid, although they were still susceptible to ciprofloxacin according to the present CLSI breakpoints. Multidrug-resistant isolates were not detected among *S. typhi *and *S. paratyphi *A in our investigation. Interestingly, 90.7% of these nalidixic resistant-isolates carried the same *gyrA *mutation, leading to the substitution Ser83Phe, which was identical to that described in Vietnam in 2007 [18]. Importantly, the incidence of *S. paratyphi *A infection has surpassed that of *S. typhi *infection since 2003 in this study. The similar results had been reported in Guangxi Autonomous Region, China [19], reinforcing our results. A disproportionate increase in the incidence of enteric fever caused by *S. paratyphi *A has also been noted in the United States, India, Nepal, Pakistan, and Thailand [20]. A report from the United States confirmed that paratyphoid fever most often was caused by nalidixic acid-resistant *S. paratyphi *A, and like typhoid fever, was usually acquired while traveling internationally. In this observation, infection with *S. paratyphi *A was associated with travel to South and Southeast Asia, and nalidixic acid-resistant infection was associated with travel to South Asia [20].

PFGE is currently the method for the subtyping of sporadic or epidemic *Salmonella *isolates. By the use of a standardized PFGE protocol in this study, the PulseNet protocol, all isolates of *S. paratyphi *A were assigned to type A, subtype A1 or A2, which suggests endemic disease from the presence of a single clone over 6-year period. By investigating 62 medical records of inpatients infected by *S. paratyphi *A, it was confirmed that five patients infected by *S. paratyphi *A had traveled to other domestic cities or regions, and one had traveled internationally to Bangladesh. Our data also suggests that the same clone of *S. paratyphi *A was present in China over the study period.

An outbreak of paratyphoid fever associated with *S. paratyphi *A in New Delhi, India was investigated by PFGE [21]. The five sporadic isolates of *S. paratyphi *A gave PFGE patterns following *Xba*I digestion that were distinct, with differences of 8 to 12 bands. In contrast, the 13 outbreak isolates shared only four closely related PFGE patterns differing only in 1 to 6 bands. Similar results were obtained after digestion with a second restriction endonuclease, *Spe*I. In another study, a total of 39 human isolates of *S. paratyphi *A from Pakistan, India, Indonesia and Malaysia were typed by PFGE using X*ba*I restriction digests. This study suggested that a limited number of clones were responsible for paratyphoid fever in those countries [22]. Similarly, the high proportion of *S. paratyphi *A infection in Nepal during 2001 was due to the emergence of a single clone [23]. In a recent report by Gupta et al [20], 110 isolates of *S. paratyphi *A were typed by PFGE of X*ba*I and *Bln*I restriction digests, which were obtained from patients with paratyphoid fever in the United States from 2005 to 2006. Thirty-one molecular subtypes (unique combinations of *Xba*I and *Bln*I patterns) were identified, and six subtypes (19%) accounted for 90 (82%) of these isolates.

## Conclusions

Nalidixic acid-resistant *S. typhi *and *S. paratyphi *A blood isolates were highly prevalent in Shenzhen, China. PEGF showed the variable genetic diversity of nalidixic acid-resistant *S. typhi *and limited genetic diversity of nalidixic acid-resistant *S. paratyphi *A that suggests a clonal expansion of *S. paratyphi *A infection in the community.

## Authors' contributions

**WW **conceived the study, participated in its design, carried out the molecular genetic studies, performed the analysis and interpretation of the data and wrote the manuscript. **HW **conceived the study, participated in its design, performed the analysis and interpretation of the data, and participated in writing the manuscript. **JL **participated in conceiving the study, its design, and interpreting the molecular data. **JW **participated in the study design and interpretation of the data. **MC **participated in the study design, analysis and interpretation of the data. **YX **participated in the study design and interpretation of the data. **YL **participated in conceiving the study. All authors have read and approved the final manuscript.
